# Assessment of brain natriuretic peptide and copeptin as correlates of blood pressure in chronic hypertensive pregnant women

**DOI:** 10.1186/s40885-022-00221-6

**Published:** 2022-12-15

**Authors:** Chika J. Okwor, Kayode S. Adedapo, Oluwasomidoyin O. Bello, Ijeoma A. Meka, Chukwuemeka V. Okwor, Chukwuemelie Z. Uche, Chiebonam E. Nwajiobi, Uloaku A. Nto-Ezimah, Chisom E. Uchechukwu, Ekene J. Arum

**Affiliations:** 1 Department of Chemical Pathology, University College Hospital, Ibadan, Nigeria; 2 Department of Obstetrics and Gynaecology, University College Hospital, Ibadan, Nigeria; 3 Department of Chemical Pathology, University of Nigeria Teaching Hospital, Enugu, Nigeria; 4 Department of Radiation Medicine, University of Nigeria Teaching Hospital, Enugu, Nigeria; 5 Department of Medical Biochemistry & Molecular Biology, University of Nigeria, Enugu, Nigeria

**Keywords:** Brain natriuretic peptide, Copeptins, Hypertension, Pregnancy

## Abstract

**Background:**

Hypertensive disorders of pregnancy including preexisting (or chronic) hypertension are the most common complication encountered during pregnancy that contribute significantly to maternal and perinatal morbidity and mortality. Brain natriuretic peptide (BNP) and copeptin have been investigated as biomarkers in various hypertensive disorders, but studies of their clinical value in chronic hypertensive pregnant women are sparce. This study aimed to assess the levels of BNP and copeptin in chronic hypertensive pregnant women and investigate their correlation with blood pressure (BP) in chronic hypertensive pregnant women in South Western Nigeria.

**Methods:**

One hundred and sixty consenting pregnant women in their third trimester of pregnancy, grouped into those with chronic hypertension (*n* = 80) and normotensive (*n* = 80), were recruited for this cross-sectional study. Age and clinical characteristics were obtained, and blood was aseptically drawn for BNP and copeptin measurement using enzyme-linked immunosorbent assay. Data was analyzed with IBM SPSS ver. 20.0. Data was analyzed using Student t-test, chi-square, and Pearson correlation test as appropriate. Statistical significance was set at *P* < 0.05.

**Results:**

The mean systolic BP (SBP) and diastolic BP (DBP) were significantly higher in pregnant women with chronic hypertension (158.30 ± 3.51 and 105.08 ± 2.47 mmHg, respectively) compared with normotensive pregnant women (100.72 ± 3.02 and 70.29 ± 1.96 mmHg, respectively). The mean levels of BNP and copeptin were higher in pregnant women with chronic hypertension (57.26 ± 3.65 pg/mL and 12.44 ± 1.02 pmol/L, respectively) compared with normotensive pregnant women (49.85 ± 2.44 pg/mL and 10.25 ± 1.50 pmol/L, respectively) though not statistically significant. Correlations observed between SBP and DBP with levels of BNP (*r* = 0.204, *P* = 0.200; *r* = 0.142, *P* = 0.478) and copeptin (*r* = − 0.058, *P* = 0.288; *r* = 0.045, *P* = 0.907) were not statistically significant.

**Conclusions:**

There was no association between BP and the levels of BNP and copeptin in pregnant women with chronic hypertension who were already on antihypertensive treatment, with the implication that antihypertensive treatment may modulate BNP and copeptin release despite significantly elevated BP levels.

## Background

Hypertensive disorders of pregnancy rank as the second most common cause of direct maternal mortality [1], with hypertension identified as the most common medical complication encountered during pregnancy [2]. Hypertensive disorders of pregnancy include preexisting (or chronic) hypertension, gestational hypertension, preeclampsia, and eclampsia. Together, these hypertensive disorders of pregnancy contribute significantly to maternal and perinatal morbidity and mortality, and account for over 30,000 global maternal deaths annually and 10 to 15% of maternal deaths in low-income and middle-income countries [3, 4].

The American College of Obstetrics and Gynecology defined chronic hypertension in pregnancy as systolic blood pressure (SBP) ≥ 140 mmHg and/or diastolic blood pressure (DBP) ≥ 90 mmHg before pregnancy or, in recognition that many women seek medical care only once pregnant, before 20 weeks of gestation, use of antihypertensive medications before pregnancy, or persistence of hypertension for > 12 weeks after delivery [5]. Chronic hypertension is estimated to affect 3% of pregnancies with an increased risk of superimposed preeclampsia among pregnant women with chronic hypertension compared to the general pregnant population [6, 7]. Adverse perinatal outcomes such as stillbirth, fetal growth restriction, and prematurity are also more common in women with chronic hypertension [8, 9].

Chronic hypertension is primary in approximately 80 to 90% of cases, and in 10 to 20% of cases, secondary to collagen vascular disease, renal, endocrine, or vascular disorders [10]. There has been a trend of increasing prevalence of chronic hypertension in pregnancy, with this increasing prevalence attributed to growing rates of obesity and delay in childbearing ages [11]. Blacks have been reported to have a higher prevalence of chronic hypertension [12] with the onset of the condition occurring at even younger ages in blacks than in Caucasians [13]. The burden of cardiovascular disease continues to be enormous in most countries, hence it is important to identify individuals at higher risk using highly sensitive and specific biomarkers in a bid to prevent morbidity or mortality more so in pregnancy with its attendant complications.

Several biomarkers have shown potential for use in risk prediction and stratification, diagnosis, prognostication, and treatment monitoring in various clinical conditions [14]. Brain natriuretic peptide (BNP) and copeptin have been previously associated with adverse pregnancy outcomes in women with hypertensive disorders of pregnancy [15, 16]. It has been shown that plasma BNP concentrations are progressively elevated with increasing severity of hypertension particularly in patients with left ventricular hypertrophy and hence, may be a marker for hypertensive ventricular hypertrophy [17]. Copeptin, on the other hand, is an important marker for identifying high-risk patients and for predicting outcomes in a variety of diseases including advanced heart failure [18], critically ill patients suffering from hemorrhagic and septic shock [19], and acute exacerbations of chronic obstructive pulmonary disease [20]. It is used as a surrogate marker for arginine vasopressin release and routine use was encouraged by the development of a suitable assay.

A previous study demonstrated that median BNP values are < 20 pm/mL and remain stable throughout normal pregnancy but are elevated in severe preeclampsia [21]. Santillan and associates also demonstrated that elevated maternal plasma copeptin is a significant predictor of preeclampsia as early as 6 weeks of pregnancy, improving care and potentially leading to the development of preventive measures [22]. Serum copeptin level in the third trimester was also shown to predict preeclampsia and its elevation was associated with adverse perinatal outcome [23]. In these previous studies, the levels of these potential biomarkers were not investigated in relation to chronic or preexisting hypertension in pregnancy to understand how sustained management of hypertension prior to or during pregnancy may affect their levels.

This present study was aimed at assessing levels of BNP and copeptin in chronic hypertensive pregnant women and to investigate the correlation between blood pressure (BP) and the levels of BNP and copeptin in chronic hypertensive pregnant women in South Western Nigeria.

## Methods

### Participants

This cross-sectional study was conducted at the antenatal clinic of the University College Hospital, Ibadan, Nigeria and involved pregnant women in their third trimester of pregnancy (27–40 weeks gestational age). This study was approved by the Research Ethics Committee of the University of Ibadan/University College Hospital (No. UI/EC/16/0452). Written informed consent was also obtained from study participants before inclusion in this study.

One hundred and sixty consenting pregnant women aged 18 years and above were recruited over a 7-month period between June and December 2017. They comprised of 80 pregnant women with chronic hypertension and 80 normotensive pregnant women. Pregnant women with congestive heart failure, stroke, known history of renal insufficiency, smoking, thyrotoxicosis, or evidence of superimposed preeclampsia at time of enrollment were excluded from this study. Pregnant women in the third trimester of pregnancy attending the antenatal clinic at the University College Hospital, Ibadan, Nigeria were consecutively recruited for this study. An attending nurse took their BP reading, and an attending physician obtained their clinical history, paying particular attention to history of hypertension or any other hypertensive disorder. Pregnant women with known history of hypertension and/or antihypertensive drug use were recruited into the chronic hypertension group where as pregnant women with no history of hypertension and BP reading < 140/90 mmHg were recruited into the normotensive group. BP was measured using a mercury sphygmomanometer (Shanghai Healthcare International Trading, Shanghai, China) with the participant seated in a comfortable position and the arm resting at the level of the heart. The American College of Obstetrics and Gynecology recommends that in women with chronic hypertension in pregnancy who are on antihypertensive therapy, BP should be maintained between 120/80 mmHg and 160/105 mmHg. BP 160/105 mmHg was used as the cutoff to classify chronic hypertensive pregnant women with BP < 160/105 mmHg as good BP control and those ≥160/105 mmHg as poor BP control.

### Sample collection and analysis

A total of 6 mL of blood was drawn from each participant after obtaining written informed consent. Of the 6 mL, 2 mL was carefully dispensed into a plastic ethylenediaminetetraacetic acid (EDTA) bottle (for BNP estimation), 2 mL into a plain bottle (for copeptin estimation), and 2 mL was dispensed into a lithium heparin bottle (for creatinine estimation). Blood specimen bottles were transported to the laboratory immediately, centrifuged at 3000 g for 15 minutes, and plasma and serum respectively were obtained. In addition, 5 mL of random midstream urine was collected for urinalysis using dipstick method (Rapid Labs, Colchester, UK). Plasma and serum samples were stored at − 20 °C before analysis. Serum copeptin and plasma BNP levels were measured using enzyme-linked immunosorbent assay (Elabscience Biotechnology, Houston, TX, USA) while estimation of serum creatinine was done using the creatininase enzymatic method.

### Statistical analysis

Statistical analysis was done using IBM SPSS ver. 20.0 (IBM Corp., Armonk, NY, USA) and data was presented as mean ± standard error of the mean. Student t-test was used for mean comparison of variables between groups. Chi-square and Pearson correlation were used to test the association between qualitative and quantitative variables respectively. A *P*-value less than 0.05 (two-tailed) was considered to be statistically significant.

## Results

History of hypertension in the chronic hypertensive pregnant women dated back to an average of 2 years prior to pregnancy and they had all been on antihypertensive drugs (Table 1). None of the participants had a history of gestational diabetes mellitus or stroke prior to index pregnancy, nor history of smoking. One participant (1.3%) had a history of excessive sweating; however, other features were not in keeping with thyroid dysfunction. Thirty-seven women (23%) had proteinuria ranging from trace to one plus, but these were likely transient as the test read negative at a subsequent repeat 1 week later (Table 1).

**Table 1:** Clinical characteristics of chronic hypertensive and normotensive pregnant women

Characteristic	Chronic hypertensive	Normotensive	*P*-value
History of hypertension	80 (100.0)	–	< 0.001^*^
1–2 Yr before pregnancy	26 (32.5)	*–*	
> 2 Yr before pregnancy	54 (67.5)	*–*	
Antihypertensive use	80 (100.0)	–	< 0.001^*^
History of leg swelling	1 (1.3)	–	0.316
Known diabetic	–	–	> 0.999
Gestational diabetes mellitus	–	–	> 0.999
History of stroke	–	–	> 0.999
Excessive sweating	1 (2.0)	–	0.316
Smoking	–	–	> 0.999
Urinary protein	36 (45.0)	1 (1.3)	< 0.001^*^
Trace	9 (11.3)	1 (1.3)	
+	27 (33.7)	–	

^*^P < 0.05

The mean ages were 34.51 ± 0.72 years and 32.87 ± 0.88 years for chronic hypertensive and normotensive pregnant women, respectively (*P* = 0.152) (Table 2). The mean SBP and DBP were significantly higher in women with chronic hypertension compared to normotensive pregnant women (*P* < 0.05) (Table 2). There was no statistically significant difference in the mean levels of creatinine in chronic hypertensive compared with normotensive pregnant women (*P* = 0.203) (Table 2). The mean levels of BNP and copeptin were higher in pregnant women with chronic hypertension compared to normotensive pregnant women though not statistically significant (*P* = 0.093 and *P* = 0.230, respectively) (Table 2).

**Table 2:** Comparisons of mean values of chronic hypertensive pregnant women with normotensive pregnant women

Variable	Chronic hypertensive	Normotensive	*P*-value
Age (yr)	34.51 ± 0.72	32.87 ± 0.88	0.152
Gestational age (wk)	32.80 ± 0.50	31.96 ± 0.43	0.205
Systolic blood pressure (mmHg)	158.30 ± 3.51	100.72 ± 3.02	< 0.001^*^
Diastolic blood pressure (mmHg)	105.08 ± 2.47	70.29 ± 1.96	< 0.001^*^
Creatinine (mg/dL)	0.85 ± 0.07	0.74 ± 0.05	0.203
Copeptin (pmol/L)	12.44 ± 1.02	10.25 ± 1.50	0.230
Brain natriuretic peptide (pg/mL)	57.26 ± 3.65	49.85 ± 2.44	0.093

^*^P < 0.05

Thirty-eight women (47.5%) with chronic hypertension had BP ≥ 160/105 mmHg (Table 3). Comparison of the mean levels of creatinine, copeptin, and BNP showed no significant difference between chronic hypertensive pregnant women with BP ≥ 160/105 mmHg compared to chronic hypertensive pregnant women with BP < 160/105 mmHg (Table 4). There was a statistically insignificant positive correlation between SBP and DBP with levels of BNP (*r* = 0.204, *P* = 0.200; *r* = 0.142, *P* = 0.478) (Table 5). There was a statistically insignificant negative correlation between SBP with levels of copeptin (*r* = − 0.058, *P* = 0.288) (Table 5) and a statistically insignificant positive correlation between DBP with levels of copeptin (*r* = 0.045, *P* = 0.907) (Table 5).

**Table 3:** Classification of blood pressure of chronic hypertensive pregnant women based on American College of Obstetrics and Gynecology recommended blood pressure target

Blood pressure level (mmHg)	Frequency (%)
< 160/105	42 (52.5)
≥160/105	38 (47.5)

**Table 4:** Comparison of biochemical parameters between chronic hypertensive pregnant women with BP ≥ 160/105 mmHg and those with BP < 160/ 105 mmHg

Variable	BP ≥ 160/105 mmHg	BP < 160/105 mmHg	*P*-value
Creatinine (mg/dL)	0.81 ± 0.05	0.75 ± 0.06	0.405
Copeptin (pmol/L)	12.15 ± 0.91	11.88 ± 1.00	0.843
Brain natriuretic peptide (pg/mL)	59.48 ± 3.34	55.94 ± 4.21	0.518

*BP* Blood pressure

**Table 5:** Correlation of blood pressure with serum copeptin and plasma brain natriuretic peptide of participants

Variable	Chronic hypertensive		Normotensive	
	SBP	DBP	SBP	DBP
Brain natriuretic peptide				
*r*	0.204	0.142	0.165	−0.060
*P*-value	0.200	0.478	0.352	0.774
Copeptin				
*r*	−0.058	0.045	0.297	0.198
*P*-value	0.288	0.907	0.076	0.300

*SBP* systolic blood pressure, *DBP* Diastolic blood pressure

## Discussion

Pregnancy has been associated with substantial decrease in peripheral resistance as early as 5 weeks of gestation, with many women with prepregnancy chronic hypertension reported to have normalization in their BP independent of antihypertensive treatment [24]. However, there is evidence of raised BP (SBP > 140 mmHg and/or DBP > 90 mmHg) among chronic hypertensive pregnant women on antihypertensive medication [25]. This present study showed significantly higher SBP and DBP in pregnant women with chronic hypertension compared with controls. This supports the findings of Nzelu et al. [25] who reported elevated BP in 28.5% of the chronic hypertensive pregnant women studied. Increased BP observed in these pregnant women puts them at significant risk of development of severe hypertension and other pregnancy complications. Hence, there is a need for close monitoring of BP levels in pregnant women with chronic hypertension in a bid to foster proper BP control.

It is recommended that in women with chronic hypertension in pregnancy who are on antihypertensive therapy, BP should be maintained between 120/80 mmHg and 160/105 mmHg [5], However, this study revealed that about 47.5% had BP ≥ 160/105 mmHg despite antihypertensive treatment suggesting poor control of BP. The reason for this is unclear as all the women indicated compliance with medications. A possible explanation is that genetic factors or influences may play a role in the suboptimal response to antihypertensive treatment in this environment, although poor compliance may not be completely ruled out. Furthermore, poor BP control observed could also be due to the type and class of antihypertensive medication used by these women. This also buttresses the need for personalized care to achieve more efficient management of medical conditions.

There was no statistically significant difference in the levels of plasma BNP between chronic hypertensives and normotensive pregnant women. This agrees with the study by Whitcomb et al. [26] who found that BNP levels were equivalent between normal and chronic hypertensives. This observation may be explained by the fact that these women have been on antihypertensive medications which possibly have modulatory effects on BNP release. Again, chronic hypertensives without superimposed preeclampsia would most likely not have acute stress to the heart, hence no significant increase in BNP release as demonstrated in a previous study [27]. Also, no significant difference in mean plasma copeptin levels was observed between women with chronic hypertensive and the normotensive controls. The pathophysiology is unclear but modulation of the hypothalamic-pituitary-adrenal axis by antihypertensive agents could be a factor. The hypothalamic-pituitary-adrenal axis has been suggested as one of the mediators of the association between copeptin and hypertensive disorders [28].

We observed that there was no statistically significant correlation between the BP levels and plasma BNP levels in women with chronic hypertension. This disagrees with the findings of Estrada et al. [29] and Eguchi et al. [30], who found a significant positive correlation between BP levels and BNP levels, and proposed that BNP levels may be used as a marker of BP control. In this study, patients with chronic hypertension who were on antihypertensives did not show elevated BNP levels as would be expected for their levels of BP which does not support the potential clinical application of BNP in the monitoring of the adequacy of treatment as demonstrated in a previous study [29]. This finding suggests that antihypertensive treatment may possibly have modulatory effect on BNP release, and is in agreement with the findings of Esunge et al. [31] as well as Resnik et al. [21] who demonstrated that antihypertensive treatment lowers plasma BNP levels. The fact that some antihypertensive medications may cause neurohormonal modification, left ventricular ejection fraction improvement, arrhythmia prevention, and ventricular rate control [32], which may culminate in reduction of cardiac wall stretch thereby reducing BNP release, may proffer an explanation for this observation.

This study has implications for the clinical management of chronic hypertensive pregnant women. It demonstrates that third trimester levels of BNP and copeptin in chronic hypertensive pregnant women already on antihypertensive medication are not indicative of BP control nor do they distinguish the chronic hypertensive pregnant women from normotensive pregnant women.

## Conclusions

There was no association between BP and the levels of BNP and copeptin in pregnant women with chronic hypertension who were already on antihypertensive treatment, with the implication that antihypertensive treatment may modulate BNP and copeptin release despite significantly elevated BP levels.

This study has significant limitations including its small sample size and the cross-sectional study design. A longitudinal study design would have allowed the authors assess pregnancy outcomes and its associations both with the levels of BNP and copeptin as well as the third trimester BP levels. In addition, patient’s self-report of antihypertensive medication compliance may have introduced some recall bias. Future studies with a larger population are encouraged to validate these findings as well as to unravel the pathophysiology and possible genetic mechanisms behind the association between the levels of BNP and copeptin with BP control in pregnant women with chronic hypertension.

## Data Availability

Not applicable.

## Abbreviations

BP: Blood pressure
BNP: Brain natriuretic peptide
DBP: Diastolic blood pressure
SBP: Systolic blood pressure
